# Case Report: Non-reactive vasoreactivity testing in a patient with patent ductus arteriosus with pulmonary hypertension: is there still a way to turn things around?

**DOI:** 10.3389/fcvm.2025.1569963

**Published:** 2025-08-05

**Authors:** Sisca Natalia Siagian, Elsa Hedia Panjaitan, Pandu Prasetyo Nugroho

**Affiliations:** ^1^ Division of Pediatric Cardiology and Congenital Heart Disease, Department of Cardiology and Vascular Medicine, Faculty of Medicine Universitas Indonesia/National Cardiovascular Centre Harapan Kita, Jakarta, Indonesia; ^2^ Faculty of Medicine Universitas Indonesia, Jakarta, Indonesia

**Keywords:** patent ductus arteriosus, pulmonary hypertension, acute vasoreactivity testing, closure test, sildenafil, percutaneous closure

## Abstract

**Background:**

Recent guidelines recommend patent ductus arteriosus (PDA) closure in adults based on hemodynamic criteria, such as pulmonary vascular resistance (PVR) and flow ratio (Qp:Qs). However, additional parameters like acute vasoreactivity testing (AVT) and closure testing, though lacking extensive data, may assist in identifying patients eligible for closure. We present the case of an adult patient with PDA and pulmonary hypertension (PH) whosuccessfully underwent transcatheter device closure guided by AVT and closure testing.

**Case presentation:**

A 35-year-old female presented with a two-year history of shortness of breath, cyanosis, and peripheral edema. She had been diagnosed with PDA at birth but did not undergo ligation due to parental refusal, leading to a 33-year loss of follow-up. Echocardiography revealed a bidirectional shunt through the PDA. Following cardiac catheterization (Qp:Qs 1.38, PVR: 21.5 WU) with AVT, the patient was diagnosed with PDA, PH with low flow, high resistance, and non-reactive to vasoreactivity test. She was prescribed sildenafil and discharged. After one year, the patient reported symptom improvement, with repeat catheterization showing a slight reduction in Qp:Qs (1.25) and PVR (16.38 WU), though values remained above the guideline cut-off for closure. However, the patient was then reactive to AVT, so we decided to perform device closure and observed the patient for 10 min before releasing the device. The patient was stable following the procedure and recovered well. One month later, the patient experienced significant symptom relief and could engage in moderate physical activity without discomfort.

**Conclusion:**

This case highlights the potential AVT, closure testing, and also the treat-and-repair strategy with sildenafil to expand the window of operability in adult PDA patients with PH. Further research especially on long-term outcomes, is recommended.

## 1 Introduction

Patent ductus arteriosus (PDA) is mostly diagnosed and treated during infancy and childhood, however, it is not uncommon to be treated only in adulthood, especially in developing countries with more limited healthcare resources. PDA that remains untreated into adulthood usually develops complications such as left ventricular (LV) volume overload and pulmonary hypertension (PH), making treatment more challenging and closure less feasible. According to the European Society of Cardiology (ESC) 2020 guideline, PDA closure in adult patients are based on hemodynamic measurements such as pulmonary vascular resistance (PVR) and flow ratio (Qp:Qs) (1). Patients who do not fit these criteria only receive palliative therapy and therefore may have worse prognoses. Other parameters, such as vasoreactivity test, closure test, and lung biopsy have the potential to be considerations in determining the feasibility of defect closure, which may enable more patients still eligible for defect closure to receive appropriate therapy. However, current evidence supporting these methods is limited.

In this report, we present a case of an adult patient with PDA with PH who was outside the criteria for closure based on current guidelines but successfully underwent transcatheter device closure based on considerations from vasoreactivity testing and closure test.

## 2 Case description

A 35-year-old woman was referred with the chief complaint of shortness of breath since 2 years before admission. She has already been diagnosed with PDA at birth and subsequently experienced repeated episodes of respiratory tract infections, feeding difficulties, and failure to thrive in her childhood years. She was recommended to undergo PDA ligation, but her parents refused surgery and the patient was lost to follow up for 33 years. She started experiencing dyspnea on exertion, fatigue, bluish extremities, and repeated bouts of peripheral edema 2 years ago but did not seek treatment. Her symptoms worsened over the year and she was admitted to our hospital. Her physical examination revealed a heart rate of 77 beats per min, a respiratory rate of 20 breaths per min, a room oxygen saturation of 97% on her right hand, and a room oxygen saturation of 90% on her left foot. Her first and second heart sounds were audible during auscultation, with a loud P2 component, with no murmur or gallop. Auscultation of her lungs also showed vesicular breath sounds on all chest area and no rales was audible. Echocardiography examination showed bidirectional shunt PDA, mild tricuspid regurgitation, TVG of 90 mmHg, TAPSE 2.2 mm, and an ejection fraction of 69%.

We decided to perform heart catheterization using retrograde and anterograde approaches under local anesthesia. Hemodynamic measurements obtained were as follows: mPAP 101; mAoP 101; Qp:Qs 1.38; PVR: 21.5 WU; PVRi: 36.13 WU m^2^; PVR/SVR: 0.67. Meanwhile, saturation measurements were as follows: LPA: 75%; LV: 95%; AoD: 92%. Acute vasoreactivity test using 100% FiO2 for 10 min was performed, and the results were as follows: mPAP: 101; mAoP: 102; Qp:Qs: 2.07; PVR: 8.8 WU; PVRi: 14.88 WU m^2^; PVR/SVR: 0.44. Saturation measurements were as follows: LPA: 89%; LV: 100%; AoD: 100%. The patient was diagnosed with PDA bidirectional shunt, dominant right to left shunt, pulmonary hypertension with low flow, high resistance, and non-reactive to vasoreactivity test based on our center's criteria. The patient was prescribed sildenafil 20 mg orally three times daily and discharged.

After 1 year of pharmacological therapy with sildenafil, the patient returned to our hospital for a repeat catheterization. The patient reported an improvement in complaints of dyspnea and fatigue. Her physical examination showed blood pressure of 96/60 mmHg, heart rate of 68 beats per min, respiratory rate of 16 breaths per min, and room air oxygen saturation of 96%. First and second heart sounds were regular on auscultation with no gallops or murmurs. Echocardiography showed the same findings as before which is a bidirectional shunt in the PDA with PH.

**Figure 1:**
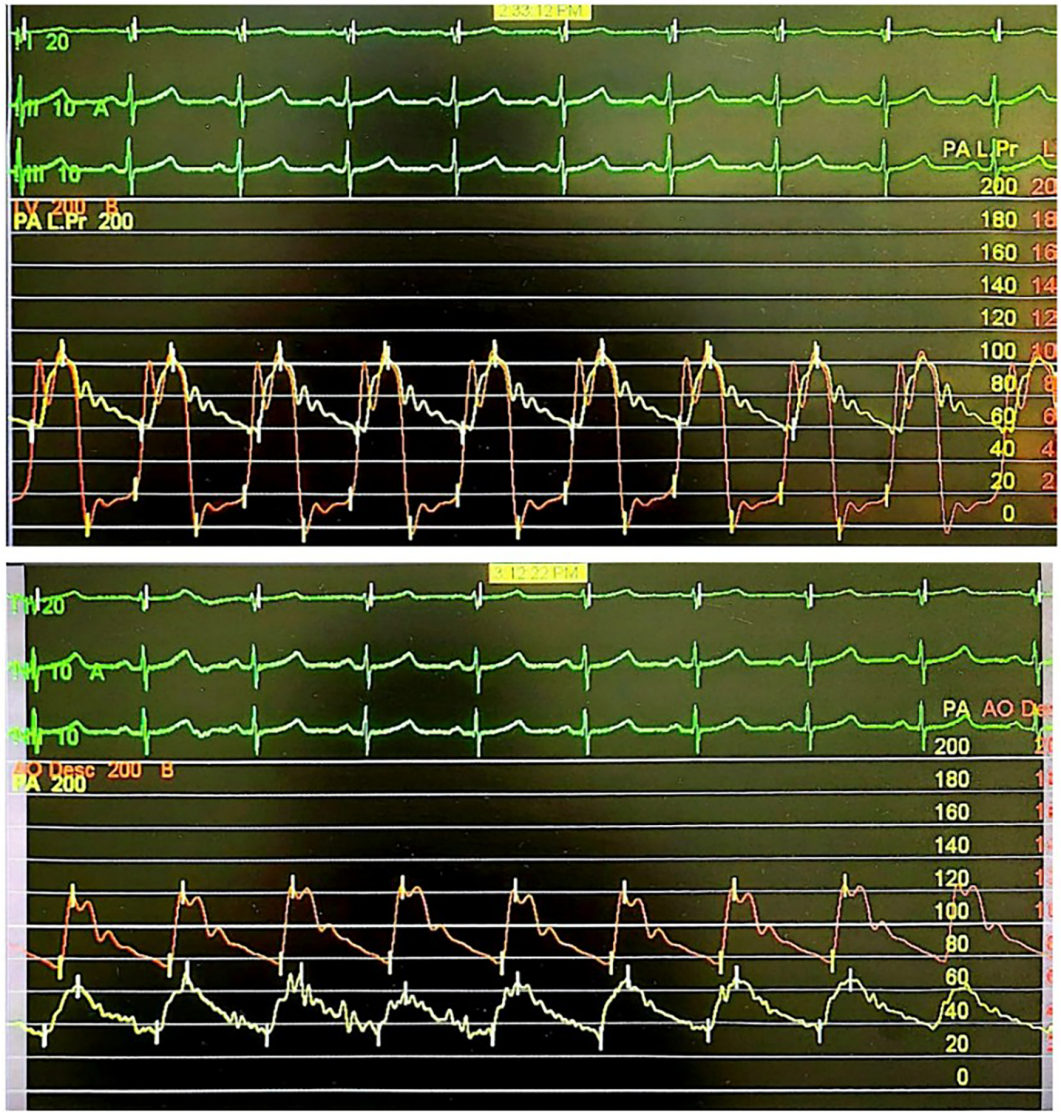
Hemodynamic measurement of the second right heart catheterization.

We decided to re-perform right heart catheterization to measure the patient's hemodynamic profile and proceed with device PDA closure if the measurements were satisfactory. Prior to the procedure, the patient had received a detailed explanation of the risks, benefits, and rationale for the planned intervention, and she agreed to proceed with the management plan. TTE/TEE examination before the catheterization revealed a type A PDA with a bidirectional shunt, isthmus 8–9 mm, ampulla 14 mm, short diastolic flow, pressure gradient 22 mmHg, mild mitral regurgitation, and mild tricuspid regurgitation. The PDA was considered suitable for device closure. Right heart catheterization was done with both anterograde and retrograde approaches, and the hemodynamic measurements obtained were as follows: mPAP: 79; mAoP: 85; Qp:Qs: 1.25; PVR: 16.38 WU; PVRi: 27.68 WU m^2^; PVR/SVR: 0.68 (Figure 1). Saturation measurements were as follows: LPA: 73%; LV: 94%; AoD: 95%. This measurement showed a slight improvement in pressure and vascular resistance compared to the previous catheterization, but the patient was still classified as having severe PH. We proceeded with an acute vasoreactivity test using FiO2 100% for 10 min, and the results were as follows: Qp:Qs: 8.21; PVR: 1.74 WU; PVRi: 2.94 WU m^2^; PVR/SVR: 0.10. Saturation measurements were as follows: LPA: 96%; LV: 96%; AoD: 97%. From this measurement, we confirmed the diagnosis of PH with low flow and high resistance, but this time the patient was reactive to the vasoreactivity test. Therefore, the decision was made to close the PDA using a MemoPart PDA Occluder No. 20/22 mm through an antegrade transvenous approach with TEE & minimal fluoroscopy guidance. The patient was then observed for 10 min to assess response to shunt occlusion and anticipate for complications due to the PH. Continuous monitoring included vital signs, PA pressure, AoD pressure, and electrocardiography (ECG). During this observation period, no significant changes occured. TTE evaluation showed appropriate device position, residual central mild shunt (+), and no obstruction in the LPA and AoD. It was decided to release the device. Post-procedure AoD pressure measurement was 122/78 (96), mPA pressure measurement was 66/34 (49) mmHg. The patient's clinical status was stable following the procedure. She recovered well and reported significant improvement in symptoms immediately after the procedure. She was discharged with a continued prescription for sildenafil.

**Figure 2:**
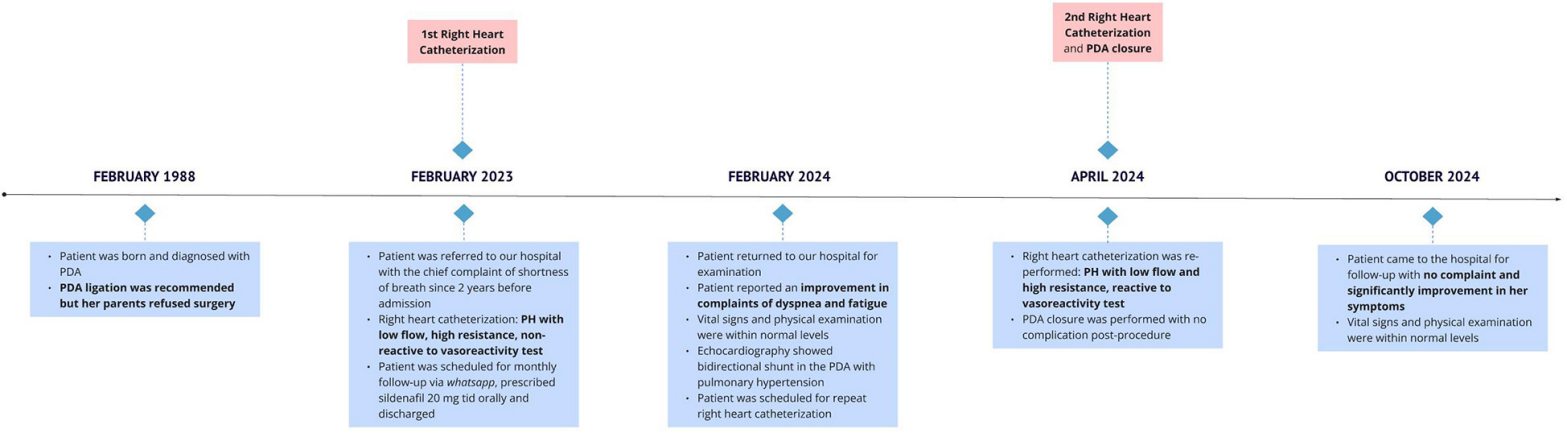
Timeline of patient's case.

One month and 6 months after the procedure, she came to the clinic for a follow-up with no complaint. She was able to participate in moderately intensive activities without any discomfort. Her room oxygen saturation is 95% and other vital signs are normal. Figure 2 illustrates the timeline of this patient's case.

## 3 Discussion

### 3.1 Patent ductus arteriosus in adults

Patent ductus arteriosus (PDA) is a congenital condition where ductus arteriosus remains open after 3 months in term infants or 1 year in premature infants (2). In some cases, including our patient's, PDA may go untreated into adulthood where itcan develop into serious complications, with some of the most common being pulmonary hypertension (PH) and heart failure. The persistent left-to-right shunt between the aorta and pulmonary artery causes increased pulmonary blood flow, which over time can elevate pulmonary vascular resistance (PVR), ultimately leading to PH. PH in congenital heart disease (CHD) is classified as pre-capillary PH, also called pulmonary arterial hypertension (PAH), defined as mean PAP >20 mmHg at rest and PVR ≥3 Wood units (WU). PDA closure is necessary to prevent further damage to the pulmonary vasculature; however, closure becomes less feasible with increasing PVR due to the associated hemodynamic changes, such as the risk of acute right heart failure in a hypertrophied and dysfunctional right ventricle, the risk of PH crisis, increased surgical risk, and even becomes contraindicated if shunt reversal has already occurred, i.e Eisenmenger's syndrome (1–3).

In our patient, PAH had progressed significantly, with measurements showing mPAP reaching 101 mmHg and PVR at 21.5 WU before the administration of sildenafil, which then became 79 mmHg and 16.38 after one year of sildenafil treatment. This creates a dilemma in determining whether the patient's PDA is still eligible for closure. Such late presentations with advanced PH are common in our center due to geosocioeconomic limitations. Because palliative medical therapy alone rarely yields satisfying results, we try to adapt our approach to identify patients who may still benefit from defect closure, even if they fall outside currently established recommendations.

### 3.2 Role of acute vasoreactivity testing in management of adult patent ductus arteriosus with pulmonary hypertension

**Figure 3:**
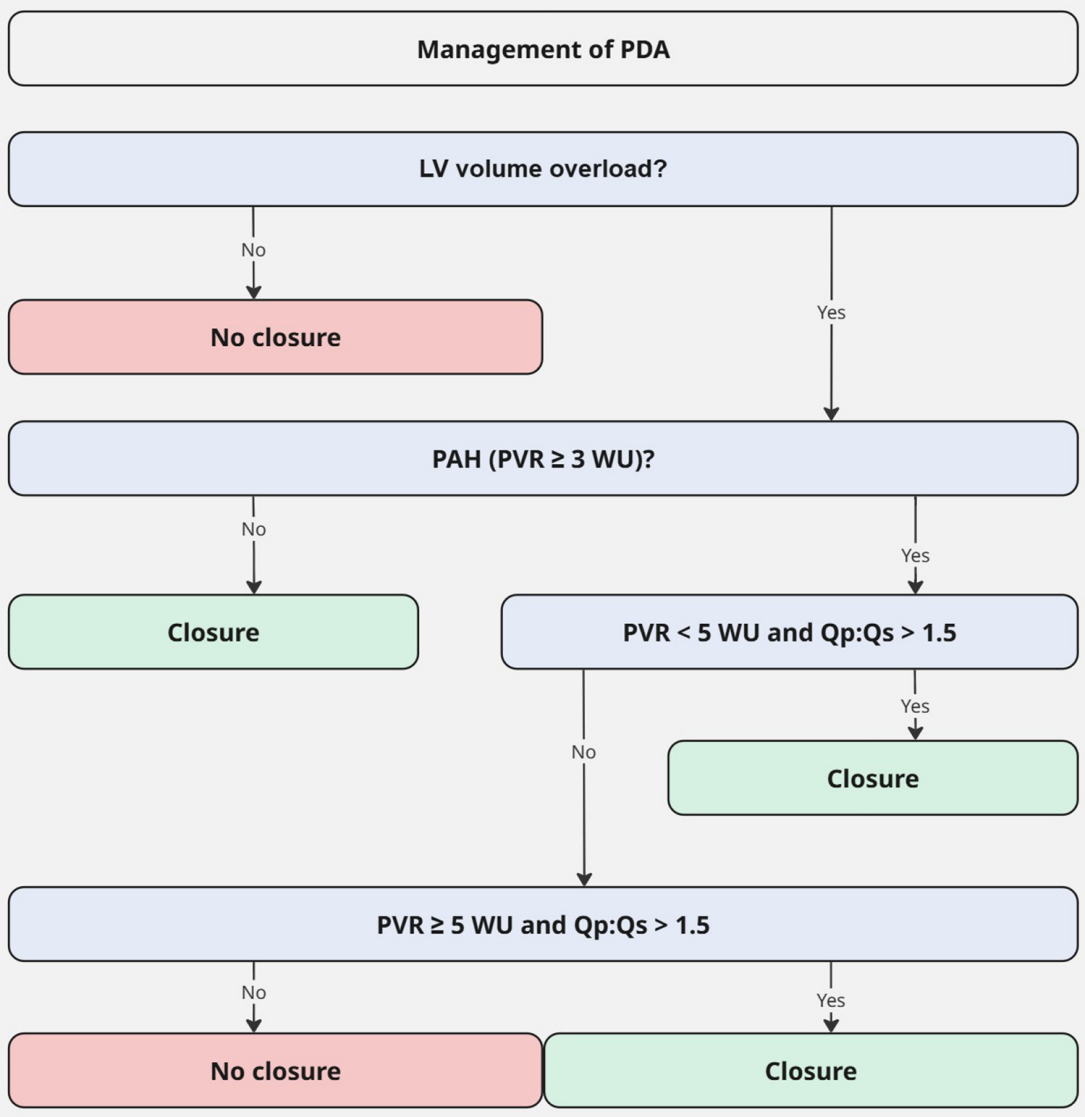
Management of PDA (adapted from 2020 ESC guideline for grown-up congenital heart disease) (1). LV, left ventricle; Qp:Qs, pulmonary to systemic flow ratio; PAH, pulmonary artery hypertension; PDA, patent ductus arteriosus; PVR, pulmonary vascular resistance; WU, Wood units.

For the management of adult PDA, the latest 2020 ESC guideline for Grown-Up Congenital Heart Disease (GUCH) recommends PDA closure for patients with LV volume overload and a PVR < 3 WU regardless of symptoms. PDA closure should also be considered in patients with PVR: 3–5 WU and may be considered in patients with PVR ≥ 5 WU, if there is still significant left-to-right shunting (Qp:Qs > 1.5). Patients with PVR ≥ 5 WU without significant left-to-right shunt or Eisenmenger syndrome are contraindicated for closure (Figure 3) (1).

Another indicator in cardiac catheterization that has been considered to assess suitability for defect closure in patients with PH-CHD is acute vasoreactivity testing (AVT). AVT is used to distinguish between reversible and progressive PAH, and thus potential operability. The definition of a positive AVT remains a controversy. Several criteria that have been used to assess response to AVT are listed in Table 1. The Barst criteria are often used for pediatric patients, while the Sitbon criteria are currently recommended for adult patients (4). In 2016, the European Paediatric Pulmonary Vascular Disease (PVD) Network proposed a modified Barst criteria, which separates the definition of a positive AVT result depending on whether prognosis or PH therapy is assessed, or operability (5). However, these hemodynamic cutoffs have not been proven sufficiently accurate to predict reversal post-shunt correction (6, 7). At our center, we use an institution-specific adaptation of vasoreactivity criteria that considers multiple hemodynamic parameters to guide clinical decision-making, particularly in patients with CHD-associated PH. Our assessment also refers to the ESC/ERS Guidelines for the diagnosis and treatment of pulmonary hypertension, which outline thresholds used to assess feasibility of shunt closure. Specifically, we define a positive AVT response as meeting the following criteria:

1. Pulmonary arterial resistance index (PARi) <8 WU·m²,
2. PVR/SVR ratio <0.33, and
3. A reduction of mPAP by more than 50% from baseline.

**Table 1:** Acute vasoreactivity test criteria.

Name	Year	Criteria for vasoreactivity
Barst	1986	• mPAP reduction ≥20% • Increase or no change in cardiac index • Decrease or no change in PVR/SVR
Rich	1992	• mPAP and PVR reduction ≥20%
Sitbon	2005	• mPAP reduction ≥10% • mPAP ≤40 mmHg • Increase or no change in cardiac output
Modified Barst	2016	• IPAH/HPAH without shunt: mPAP and PVRi/SVRi reduction >20% without decrease in cardiac output • APAH-CHD and shunt: reduction in PVRi and PVRi/SVRi >20%, PCRi <6 WU, PVRi/SVRi <0.3

mPAP, mean pulmonary arterial pressure; PVR, pulmonary vascular resistance; SVR, systemic vascular resistance; IPAH/HPAH, idiopathic/hereditary pulmonary arterial hypertension; PVRi, pulmonary vascular resistance index; SVRi, systemic vascular resistance index; APAH-CHD, pulmonary arterial hypertension associated with congenital heart disease.

AVT is currently not a recommendation in the ESC GUCH guidelines as it is considered to not yet have sufficient data. Still, some previous studies show the potential of AVT in selecting PH-CHD patients who may benefit from defect closure. The 2015 AHA/ATS guideline also recommends the use of AVT; however, this guideline is intended for pediatric patients (8). Repeated AVT testing is also not routinely performed, despite some patients showing an increase in pulmonary vasoreactivity on their second RHC after undergoing PAH treatment (9).

In our patient, we performed the first AVT with a negative result, then gave the patient targeted drug therapy with sildenafil for one year, and then performed a repeat AVT with a positive result, which was the basis for proceeding with device closure. Based on experience in our center, including with this patient, AVT is useful in assessing the suitability of defect closure for simple shunt lesions including in adult patients, with considerations of other clinical factors, supporting examinations, and hemodynamic measurements of the patient. We suggest that AVT still has the potential to expand the window of operability for PDA patients with PH, so further research on AVT especially regarding the long-term outcome is worthwhile and needed.

### 3.3 Other strategies for patent ductus arteriosus complicated with pulmonary hypertension

The strategy that we applied to our patient of administering medication to improve the patient's PH, allowing for defect closure is commonly referred to as the “treat-and-repair” strategy (10, 11). For patients initially ineligible for shunt closure, oral endothelin receptor antagonists (ERA) or phosphodiesterase 5 inhibitors (PDE5i) therapy are typically used. Bosentan has been shown to improve the 6-minute walking test (6MWT) and decrease PVR in patients with Eisenmenger syndrome and WHO functional class III, although evidence regarding its effect on mortality remains limited. Sildenafil and tadalafil also demonstrated beneficial effects on functional and hemodynamic parameters, improving exercise capacity and hemodynamics. Long-term sildenafil therapy has been associated with improved or stable WHO functional class and is generally well tolerated. Furthermore, sildenafil as monotherapy or combined with ERA has shown an effect in reducing PVR (12–15).

There is currently no consensus on the optimal PH-specific therapy regimen after defect closure. As demonstrated in this case, we suggest continuation of long-term treatment to reduce the risk of PH developing post-closure, which is linked to poor outcomes (16). Therapy should be individualized based on post-closure monitoring. Further research is needed to clarify the role and duration of PH-specific medications in this context.

In addition to AVT and the “treat-and-repair” strategy, we also performed a trial occlusion for 10 min to further ensure the safety of the device closure in our patient. Several recent studies have shown the benefits of the trial occlusion strategy to assess operability and prognosis in PDA patients with severe PAH, thereby potentially expanding the opportunities for patients with severe PAH to undergo repair, even though this strategy has not yet been recommended in the ESC GUCH guidelines. Trial occlusion may be an advantage of transcatheter defect closure compared to surgery because the operator can assess the patient's response in real time and can quickly withdraw the device if the patient shows a negative response to the defect closure (17–19).

While the clinical outcome in this case was favorable, it is important to emphasize that the decision to proceed with defect closure in complex cases with PH must be made with careful consideration and thorough preparation. Potential complications—such as PH crisis, pulmonary hemorrhage, and right ventricular (RV) failure—must be anticipated. This includes being prepared to remove the closure device if the patient shows signs of clinical deterioration, as well as anticipating the need for advanced supportive measures such as sedation, mechanical ventilation, administration of pulmonary vasodilators (e.g., inhaled nitric oxide, sildenafil, iloprost), inotropic support, and hemostatic agents in the event of bleeding.

## 4 Conclusion

Current guidelines dictate that PAH patients with negative AVT are contraindicated for shunt closure and are given palliative PDE5i treatment to delay the progression of the disease. Our experience showed that treatment with sildenafil led to an increase in vasoreactivity on repeat AVT and subsequent shunt closure resulted in marked improvement in symptoms.

## Data Availability

The original contributions presented in the study are included in the article/Supplementary Material, further inquiries can be directed to the corresponding author/s.
